# Septic arthritis in Iceland 1990–2002: increasing incidence due to iatrogenic infections

**DOI:** 10.1136/ard.2007.077131

**Published:** 2007-09-27

**Authors:** Á J Geirsson, S Statkevicius, A Víkingsson

**Affiliations:** Department of Medicine, Landspitali University Hospital of Iceland

## Abstract

**Objectives:**

To assess the impact of increased number of diagnostic and therapeutic joint procedures on the incidence and type of septic arthritis (SA).

**Methods:**

All cases of SA in Iceland from 1990–2002 were identified by thorough review of the available medical information. The results of synovial fluid cultures from every microbiology department in Iceland were checked and positive culture results reviewed, as well as patient charts with a discharge diagnosis of septic arthritis (International Statistical Classification of Diseases and Related Health Problems (ICD) code M009).

**Results:**

A total of 253 cases of SA (69 children and 184 adults) were diagnosed in Iceland in 1990–2002, giving an average incidence of 7.1 cases/100 000 inhabitants. The incidence of SA increased from 4.2 cases/100 000 in 1990 to 11.0 cases/100 000 in 2002. This rise in SA was primarily observed in adults where the incidence rose by 0.61 cases/100 000 per year (p<0.001). SA was iatrogenic in 41.8% of adults and the number of iatrogenic infections increased from 2.8 cases/year in 1990–1994 to 9.0 cases/year in 1998–2002 (p<0.01). The annual number of arthroscopies increased from 430 in 1990–1994 to 2303 in 1998–2002 (p<0.001) and there was a correlation between the total usage of intra-articular drugs in Iceland and the incidence of SA (p<0.01). The frequency of post-arthroscopy SA was 0.14% and post-arthrocentesis SA 0.037%.

**Conclusions:**

The incidence of SA has increased in recent years due to an increased number of arthroscopies and joint injections. Although the frequency of SA per procedure has not changed, these results emphasise the importance of sterile technique and firm indications for joint procedures.

Septic arthritis (SA) due to bacterial infection is a serious and potentially life threatening disease that can lead to rapid destruction of the vulnerable articular hyaline cartilage and irreversible loss of joint function.^1^ ^2^ The reported incidence of septic arthritis is 2–6 cases/100 000 inhabitants.^3^^–^7 Advancing age, rheumatoid arthritis (RA), osteoarthritis, immunosuppressive therapies and diabetes mellitus have been identified as the main risk factors for idiopathic septic arthritis. Iatrogenic infection resulting from joint surgeries, arthroscopies and needle insertions into joints is another source of septic arthritis. In recent years, the number of arthroscopies has greatly increased as well as the number of joint injections due to the emergence of intra-articular viscosupplementation therapies. The risk of procedure-related SA has been estimated at 0.5–2.0% for arthroscopies^8^^–^12 and 0.005–0.0002% for joint injections.^13^^–^15 In light of the growing number of individuals with one or more risk factors for SA, the incidence would be expected to be rising. However, very few of these studies have addressed this issue and most published studies are derived from selected populations. Therefore a retrospective, nationwide study was carried out to examine the incidence, cause and characteristics of septic arthritis in Iceland over a 13-year period.

## MATERIALS AND METHODS

### Clinical setting and definition of cases

Over a 13-year period from 1 January 1990 to 31 December 2002 a nationwide computerised and manual survey for culture positive joint fluids was performed in all microbiology laboratories in Iceland. In addition, medical records and archives at all hospitals that admit patients with SA were searched for the discharge diagnosis of bacterial arthritis (M009 according to the International Statistical Classification of Diseases and Related Health Problems (ICD)-9 and ICD-10 codes).

### Data collection

Hospital medical records were reviewed and clinical and laboratory data collected in a systematic manner. Iatrogenic SA was defined as occurring within 2 weeks after arthrocentesis or arthroscopy, or within 6 months after open joint surgery. The total number of arthroscopic and arthrocentesis procedures 1990–2002 were available from computerised records at the State Social Security Institute (SSI), which is the only agency in Iceland that reimburses for ambulatory medical procedures. The total import and sales of corticosteroids for intra-articular injections (methyl prednisolone (DepoMedrol; Pfizer NewYork, NewYork, USA), betamethasone (Diprospan; Schering-Plough, Kenilworth, New Jersey, USA) and triamcinolone (Lederspan; Meda AB, Solna, Sweden)) and for hyaluronic acid preparations (Hyalgan, Artzal Astra-Zeneca, Albertslund, Denmark) were similarily obtained from the SSI. Defined daily doses (DDD) for all drugs were obtained from the World Health Organization (WHO) Collaborating Centre for Drug Statistics Methodology (WHOCC). The study was approved by the Data Protection Authorities in Iceland and the National Bioethics Committee.

### Statistical methods

The age-specific incidence of infectious arthritis during the period 1990–2002 (yearly and total) was obtained by dividing the number of cases by the mean population number for each gender in each 10-year age interval (the first 2 years and 2–9 years of life were computed separately). The age-standardised incidence was computed using the global population figure as reference. The same approach was used for obtaining the standardised values for ages 0–16 and >16 years using a truncated global population figure as reference. Regression analysis was performed for the age-standardised incidence for each gender. Linear regression was first used for finding the linear time trend and then quadratic regression was used to find whether the time trend was changing with time. The association of yearly use of intra-articular drugs (DDD/1000 inhabitants) and number of septic arthritis cases was analysed with linear regression with and without adjusting for common time trend.

The same approach was used for analysing the association of number of procedures involving joints and the number of septic arthritis cases during the period 1990–2002. The level of significance used was p<0.05. All tests were two-tailed. The χ^2^ test with Yates correction for continuity was used for comparison between two groups using SigmaStat for Windows software, V.3.11 (SigmaStat, Systat Software GmbH, Erkath, Germany).

## RESULTS

### Incidence of septic arthritis

**Figure 1:**
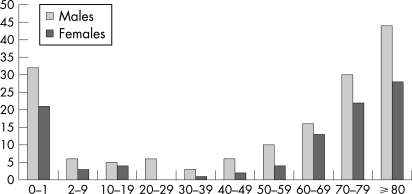
Age and gender specific incidence rates of septic arthritis for children 0–24 months, 2–9 years old and subsequent 10-year age groups.

From 1990 to 2002 a total of 253 cases of bacterial arthritis were diagnosed in Iceland, of which 159 were males and 94 females, giving an average incidence of 7.1 cases/100 000 inhabitants. Additionally, five cases of atypical mycobacterial joint infections were identified. These cases were not included in the overall analysis of SA. There were 69 children (0–16 years old) diagnosed with SA, 37 of those less than 2 years of age. Figure 1 shows age- and gender-specific incidence rates of SA for children 0–24 months old, ⩾2–10 years old and subsequent 10-year age groups. As shown in fig 1, women had lower incidence rates than men in all age groups (male/female ratio 1.7). For both sexes, the rate of SA rose sharply after the age of 50. Among children less than 2 years of age the age adjusted incidence was quite high at 27/100 000.

### Change in the incidence of septic arthritis 1990–2002

**Figure 2:**
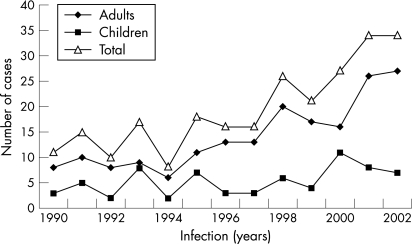
The annual total number of cases of septic arthritis for children 0–16 years and adults.

The annual total number of cases of SA for children 0–16 years and for adults are shown in fig 2. The annual incidence of SA increased progressively from 4.2 per 100 000 in 1990 to 11.0 per 100 000 inhabitants in 2002. This change was primarily due to increased number of SA cases in adults and was statistically significant by linear trend, which showed a yearly increase of 0.61 adult cases per 100 000 (p<0.001). The mean annual incidence during the first 5 years (1990–94) increased from 4.2 cases per 100 000 adult inhabitants to 9.4 per 100 000 adults in 1998–2002 (95% CI 3.2–5.5 and 7.9–11.1, respectively; p<0.01).

### Iatrogenic septic arthritis

Joint infections were iatrogenic in 1.4% (1/69 cases) of children and 41.8% (77/184 cases) of adults. Iatrogenic infections in adults were due to open joint surgery in 26 cases (14.1% of total), arthroscopy in 18 cases (9.8%) and arthrocentesis in 33 cases (17.9%) (table 1). The mean number of iatrogenic infections increased from 2.8 infections/year in 1990–1994 to 9.0 infections/year in 1998–2002 (p<0.01) and this was the main reason for the observed increase in SA during the study period. The overall percentage of iatrogenic infections was similar in young adults (age 17–65) and older adults (age ⩾65). Septic arthritis due to arthrocentesis was common in both age groups but post arthroscopy SA was primarily observed in the younger adults (15/101 vs 3/83; p = 0,02) whereas infections due to open surgery were more common in the older adults (18/83 vs 8/101; p = 0.01).

**Table 1:** Iatrogenic septic arthritis in Iceland 1990–2002

	Arthrocentesis	Arthroscopy	Open joint surgery	Total
No. of cases	33	18	26	77
Age (median)	63	47	70	60.1
Male/female ratio	2	17	1	2.1
Infected joint:				
Knee	64% (21)	89% (16)	62% (16)	69%
Shoulder	12% (4)	11% (2)	16% (4)	13%
Hip	3% (1)	0	16% (4)	6%
Other	21% (7)	0	8% (2)	12%
Synovial culture:				
Coagulase-positive Staphylococci	49% (16)	22% (4)	27% (7)	35%
Coagulase-negative Staphylococci	21% (7)	28% (5)	20% (5)	22%
Streptococci	9% (3)	11% (2)	27% (7)	16%
Other	9% (3)	17% (3)	15% (4)	13%
Negative	12% (4)	22% (4)	11% (3)	14%

Figures in parentheses are absolute no. of cases.

To determine whether the rising incidence of SA was related to increased number of diagnostic or therapeutic joint procedures, information about the total number of arthroscopies and arthrocentesis were obtained from the SSI in Iceland (table 2). The annual number of documented arthrocentesis was only available for the practice of rheumatology and orthopaedics, but not for general practitioners. We were, however, able to retrieve accurate information about the annual sales of intra-articular glucocorticoid and hyaluronan preparations for 1990–2002. There was a correlation between the usage of intra-articular betamethasone (Diprospan®; p = 0.001), hyaluronic acid (p = 0.02), and the total usage of intra-articular drugs (p = 0.008) and the incidence of SA (table 2). The number of reimbursed arthrocentesis procedures performed by rheumatologists and orthopaedists did not change markedly over the study period, but the number of arthroscopies increased from an annual mean of 430 arthroscopies in 1990–1994 to 2303 arthroscopies in 1998–2002 (p<0.001).

**Table 2:** The annual sales of intra-articular glucocorticoids and hyaluronans defined by DDD (defined daily dose) and the annual registered number of arthroscopies and arthrocentesis in Iceland

Year	Population	No. cases	Lederspan	DepoMedrol	Diprospan	Hyaloronan	Total	Arthroscopy	Arthrocentesis
1990	254 788	11	0.33	0.27	0.26	0.00	0.86	55	5829
1991	257 965	15	0.25	0.29	0.30	0.00	0.84	230	6253
1992	264 103	10	0.31	0.30	0.23	0.00	0.84	258	6808
1993	263 783	17	0.36	0.28	0.19	0.00	0.83	642	6612
1994	266 006	8	0.45	0.25	0.17	0.01	0.83	965	6404
1995	267 380	18	0.44	0.25	0.22	0.02	0.93	2245	6607
1996	268 927	16	0.46	0.24	0.20	0.07	0.97	3123	7558
1997	270 935	16	0.13	0.42	0.41	0.12	1.08	2847	8124
1998	273 764	26	0.39	0.25	0.40	0.17	1.21	1479	8297
1999	277 184	20	0.42	0.22	0.35	0.20	1.19	2157	8412
2000	281 154	27	0.40	0.22	0.28	0.22	1.12	2351	7551
2001	285 054	34	0.20	0.34	0.48	0.16	1.18	2795	7063
2002	287 559	36	0.00	0.33	0.61	0.12	1.06	2735	6891

### Clinical and microbiological characteristics of septic arthritis

The clinical characteristics of SA in children (0–2 years vs >2–16 years old) and adults (16–65 years vs >65 years old) are shown in table 3. No significant difference in joint distribution was observed between younger and older adults, although shoulder infections appeared to be more common in the elderly (13.8% vs 4.8%, p = 0.05). In adults, SA was polyarticular in 3.3% of cases.

**Table 3:** Clinical and laboratory characteristics of children and adults with septic arthritis in Iceland 1990–2002

	Children			Adults		
	<2 years	2–16 years	Total	16–65 years	>65 years	Total
No. of cases	31	38	69	101	83	184
Infected joint:						
Knee	35.5%	35%	35%	45%	53%	49%
Hip	38.7%	32.4%	35.5%	12.6%	15%	13.7%
Shoulder	6.5%	0%	2.9%	5.8%	13.8%	9.5%
Ankle	9.7%	16.2%	13.2%	10.6%	5.7%	8.4%
Hands and feet	0	7.9%	4.3%	11.9%	8.4%	10.3%
Other	9.6%	8.5%	9.0%	14.1%	4.1%	9.0%
Iatrogenic infections	0%	2.7%	1.5%	42%	40%	41.8%
Admission data:						
Temperature (°C)	38.1	38.1	38.1	38.0	38.0	38.0
Peripheral blood:						
WBC (×10^9^/litre)	13.6	10.5	11.9	11.6	9.5	10.7
ESR (mm/h)	52	28	35	54	80	68
CRP (mg/litre)	48	38	44	125	189	125
Culture positive	23%	24%	24%	28%	41%	35%
Synovial fluid:						
WBC (×10^6^/litre)	107 100	65 900	72 600	50 000	58 400	52 700
Gram stain positive	35%	35%	35%	46%	52%	49%
Culture positive	45%	62%	54%	87%	81%	84%
Normal admission values:						
Temperature <37.8°C	19%	26%	23%	41%	44%	42%
WBC <10×10^9^/litre	6%	43%	26%	36%	57%	46%
ESR <20 mm/h	8%	26%	18%	18%	7%	12%
CRP <10 mg/litre	9%	11%	7%	12%	18%	15%

Median values for WBC, ESR, CRP and temperature are shown.

CRP, C-reactive protein; ESR, erythrocyte sedimentation rate; WBC, white blood cell count.

Compared to adults the relative occurrence of SA of the hip was significantly higher in children (35.5% vs 13.7% respectively, p<0.001).

The median temperature on admission was 38–38.1°C in all age groups. Normal temperatures (<37.8°C) were quite frequent, seen in about one fourth of children and almost half of the adults (table 3). Similarily, peripheral white blood cell (WBC) count was normal (<10 000/μl) on admission in 26% of children and 46% of adults. Normal values for temperature and WBC count on admission were more common in adults (p = 0.066 and p = 0.05 respectively). Normal erythrocyte sedimentation rate (ESR) and C-reactive protein (CRP) values were infrequent and only 2.3% of children and 4.4% of adults had normal values for ESR and CRP on admission.

Synovial fluid was obtained in 97.4% of cases (98.6% of children, 97.3% of adults). Results from synovial fluid analysis are shown in table 3. Although the leukocyte count was markedly elevated (median = 58 100/mm^3^) a relatively low synovial fluid leukocyte count (<20 000/mm^3^) was observed in 7% of children and 15% of adults. Synovial fluid culture was positive in 84% of adults but only in 54% of children. Blood cultures were obtained in 96% (66/69) of children and in 74% (137/184) of adults. They were positive in one quarter to one third of individuals tested, arguably more often in the elderly (41%, p = 0.056). Positive Gram stain and/or blood culture increased the positively identified septic arthritis cases considerably, from 54% to 61% in children and from 84% to 88% in adults. The yield of synovial fluid culture was significantly lower in adult patients who had received antibiotics prior to diagnostic joint aspiration (10/27 negative, 37%) compared to patients without antibiotic exposure (15/119 negative, 12.6%; p = 0.002).

The contributing risk factors for SA among adults were history of a recent trauma to the involved joint in 24% of cases, osteoarthritis in 19%, diabetes mellitus (DM) in 7% all having type II DM and being treated with per oral hypoglycaemic drugs, rheumatoid arthritis in 4% all being treated with methotrexate, neoplasia in 2% and one patient was HIV positive. Additionally, three patients were receiving glucocorticoids. In 16 (8.7%) of the adult cases SA was due to infected prosthetic joints, 12 knees, 3 hip joints and 1 shoulder joint.

The type and frequency of bacterial organisms is shown in table 4.

**Table 4:** Results of synovial fluid culture in septic arthritis in Iceland

Bacteria	Children			Adults		
	<2 years, n = 31	2–16 years, n = 38	Total, n = 69	16–65 years, n = 101	>65 years, n = 83	Total, n = 184
*Staphylococcus aureus*	6.5% (2)	34% (13)	21.7% (15)	45% (46)	38.6% (32)	42.6% (78)
Streptococci	16% (5)	16% (6)	15.9% (11)	16% (16)	20.5% (17)	18.0% (33)
Coagulase-negative Staphylococci	6.5% (2)	0	2.9% (2)	14% (14)	8.4% (7)	11.5% (21)
Gram negative rod	3.2% (1)	2.6% (1)	2.9% (2)	4% (4)	7.2% (6)	5.5% (10)
*Kingella kingae*	6.5% (2)	5.2% (2)	5.8% (4)	0	0	0
Other	3.2% (1)	5.2% (2)	4.4% (3)	6% (6)	3.6% (3)	4.9% (9)
Culture negative	55% (17)	37% (14)	44.9% (31)	13% (13)	18.1% (15)	15.3% (28)
Culture not obtained	3.2% (1)	0	1.5% (1)	2% (2)	3.6% (3)	2.7% (5)

Figures in parentheses are absolute no. of cares.

A total of 33 of the adult cases were not identified by positive joint fluid culture, and of those, 4 had positive Gram stain of joint fluid and 4 had positive blood cultures. In all, 32 (46%) children were not identified by positive joint fluid culture, but one of those had positive Gram stain of joint fluid and three had positive blood culture. The clinical and laboratory characteristics of children and adults with SA diagnosed without positive joint fluid culture was similar to the culture positive SA. Specifically, median age, sex and joint involvement were comparable, as well as ESR, CRP and synovial fluid leukocyte count, duration of antibiotic therapy and hospital stay (data not shown).

Of the 254 patients, 5 died in the hospital, (mean age, 70 years); 3 following infection with *Staphylococcus aureus*, 1 after pneumococcal infection, and 1 following streptococcal infection. The mortality rate was 1.8% when calculated from all cases of septic arthritis but 2,7% if limited to the adult population.

From 1990–2002 we identified five cases of arthritis due to mycobacteria other than tuberculosis; two *Mycobacterium kansasii*, two *Mycobacterium avium-intracellulare* and one *Mycobacterium marinum* infection. These infections affected three elbows and two wrists joints in four men and one woman, 34–49 years old. One of these patients was HIV positive but the other four had no identifiable immunodeficiency. These mycobacterial infections were not included in the overall analysis of the data.

## DISCUSSION

In this paper we report a markedly increased rate of joint infections in adults in Iceland from 1990 to 2002. During the study period there was a mean annual increase of 0.61 cases/100 000 adult inhabitants. The incidence of 4.2 cases/100 000 adults in 1990–1994 is similar to the reported number in other studies^3^–^6^ ^16^ but the incidence of 9.4/100 000 adults during 1998–2002 is considerably higher and statistically different (p<0.01). This change is primarily due to increase in iatrogenic infections following joint arthroscopies and arthrocentesis. It coincides with marked increase in the registered number of joint arthroscopies in Iceland and increased use of intra-articular steroids and joint-viscous supplements in Iceland during these years. However, this increase in SA following joint injections was not due to a higher number of arthrocentesis procedures performed by rheumatologists and orthopaedists. However, the increased use of intra-articular steroids and joint-viscous supplements suggests that the number of arthrocentesis procedures did indeed increase during this observational period. That scenario could be explained by higher numbers of arthrocentesis procedures being performed by other doctors. This is however purely speculative, since computerised documentation of arthrocentesis in primary care is mostly unavailable. Septic arthritis due to open joint surgeries did not increase over this time period.

The frequency of post-arthroscopic SA has been reported 0.1–0.5% at specific medical centres^9^^–^12 but to our knowledge this frequency has not been previously estimated based on a nationwide analysis. The estimated frequency in Iceland of 0.14% is similar to these previous reports.

There is very limited data available in the medical literature about the actual risk of SA following arthrocentesis. In early reports this risk was estimated at 0.005 to 0.0002% per arthrocentesic procedure.^13^^–^15 These percentages are lower than can be estimated from our study. According to the SSI registry in Iceland the total number of documented ambulatory arthrocentesis in Iceland performed by rheumatologists and orthopaedists is 6900 per year (table 2). Additionally, an estimated maximum of 1000 arthrocentesis procedures are performed annually by general practitioners (L. Ólafsson, the Center of Health Care in Reykjavik,  Iceland personal communication). Accordingly, the estimated risk of septic arthritis following arthrocentesis in Iceland is 3 infections/7900 procedures, or 0.037% per injection. Compared to other commonly performed procedures, an incidence of 0.037% per injection is not high. However, given the significant morbidity and 10–15% mortality previously reported in SA^3^–^6^ ^16^ it is of utmost importance to avoid unnecessary infectious complications and to perform arthrocentesis according to the best standard of practice.

In our study 17,9% of SA in adults occurred post-arthrocentesis, which is alarmingly high compared to the frequency of 1.9–3% in previous studies.^4^ ^6^ ^7^ It has been suggested that intra-articular steroid^17^ or hyaluronan^18^ injection may increase the risk of joint infection. Thus, the generally increased use of steroids and hyaluronans in Iceland (table 2) may explain the increased incidence of SA in Iceland.

Presumably organisms frequently enter the joint during arthrocentesis. Skin fragments introduced into the joint during arthrocentesis contained bacterial genes (by polymerase chain reaction) one third of the time.^19^ and despite standard sterilisation of the skin surface, organisms could be cultured from needle tips in 14–28% of events.^20^ The best standard of practice for arthrocentesis has not been thoroughly studied and method of sterilisation varies considerably among doctors;^21^ alcohol swabs, pivodone iodine and chlorhexidine have been used in different reports, all apparently with satisfactory results, although chlorhexidine arguably has the edge over the other two methods. Chlorhexidine resulted in better sterilisation of needle tips compared to alcohol swabs^20^ and chlorhexidine use yielded significantly lower skin pathogen contamination of blood cultures compared with pivodone iodine.^22^ It may also be of practical importance in the clinical settings that full bacteriocidal effects from pivodone iodine takes over 1 min to develop.^22^ As far as we know, all three types of antiseptic techniques are in use in Iceland but no survey has been conducted to assess their prevalence among doctors. We were not able to correlate the type of antiseptic technique used to the rate of SA in this retrospective study. Irrespective of this, a panel of experts from the American College of Rheumatology emphasised that intra-articular corticosteroid injections are “safe and effective when administered by an experienced doctor”.^23^ This may indeed be the most important factor in avoiding iatrogenic SA following arthrocentesis.

The clinical characteristics of SA have been previously reported in children^24^^–^28 adults,^3^^–^6 in the elderly;^29^^–^32 and numerous reviews have been published on septic arthritis.^33^^–^36 Most previous studies in adults have been derived from selected regions or populations, although one report represented a whole communal region.^5^ The current report is the first study reporting a nationwide survey. We screened all microbiology laboratories in Iceland for positive joint fluid cultures, and all hospitals in Iceland for ICD-9 and-10 discharge diagnoses of bacterial arthritis. Our study has numerous clinical and laboratory findings similar to previous reports. Thus, the sex ratio of 1.7 (male/female), risk factors (including advancing age, RA, osteoarthritis (OA), DM, iatrogenic illness), joint involvement and pathogens cultured are quite comparable to these reports. In our study, the frequency of polyarticular involvement among adults was quite low (3.3%) compared to 8.4–19.5% in other studies^7^ ^8^ and mortality among adults was only 2.7%, considerably lower than reported in most studies,^3^^–^6 19although similar to some.^29^ Our study agrees with previous findings that markers of systemic inflammation (fever, leukocytosis, elevated ESR or CRP) can be normal on presentation in SA. In fact normal temperature, WBC count, ESR and CRP were observed in 42%, 46%, 12% and 15%, respectively, in adults (table 3). Furthermore, although SA is usually associated with high synovial leukocyte count we observed counts less than 20 000 in 15% of adult cases. Our study emphasises the importance of obtaining blood cultures as well as synovial fluid cultures, and highlights the potential for false negative results if the patient has received oral antibiotics prior to obtaining cultures. Thus, the diagnosis of SA in adults according to our study should primarily be derived from thorough medical history and physical examination, supported by joint fluid WBC analysis and confirmed by synovial/blood cultures in 88% cases. Antibiotic exposure prior to synovial culture may decrease the culture yield to 63%.

Surprisingly, 39% of children with clinical picture of septic arthritis had negative synovial fluid and blood cultures. The clinical and laboratory characteristics were similar in culture positive and negative children. These results are identical to numerous other reports^24^^–^28 and to date no reasonable explanation has been put forward.

In summary, the incidence of septic arthritis has markedly increased in recent years due to increased number of arthroscopies and therapeutic joint injections. Although the frequency of SA per procedure has not changed, these results emphasise the importance of sterile technique and firm indications for diagnostic and therapeutic joint procedures.

## References

[1] Shirtliff ME Mader JT Acute septic arthritis. Clin Microbiol Rev 2002;15:527–44 12364368 10.1128/CMR.15.4.527-544.2002 PMC126863

[2] Howard JB Highenbothen CL Nelson JD Residual effect of septic arthritis in infants and childhood. JAMA 1976;236:932–5 988890

[3] Cooper C Cawley MID Bacterial arthritis in an English health district: a 10 year review. Ann Rheum Dis 1986;45:458–63 3729573 10.1136/ard.45.6.458 PMC1001917

[4] Weston VC Jones AC Bradbury N Fawthrop F Doherty M Clinical features and outcome of septic arthritis in a single UK Health District 1982–1991. Ann Rheum Dis 1999;58:214–19 10364899 10.1136/ard.58.4.214 PMC1752863

[5] Kaandorp CJE Krijnen P Moens HJB Habbema JDF van Schaardenburg D The outcome of bacterial arthritis: a prospective community-based study. Arthritis Rheum 1997;40:884–92 9153550 10.1002/art.1780400516

[6] Gupta MN Sturrock RD Field M A prospective 2-year study of 75 patients with adult-onset septic arthritis. J Rheumatology 2001;40:24–30 10.1093/rheumatology/40.1.24 11157138

[7] Kaandorp CJE Dinant HJ van de Laar MAFJ Moens HJB Prins APA Dijkmans BAC Incidence and source of native and prosthetic joint infection: a community based prospective survey. Ann Rheum Dis 1997;56:470–5 9306869 10.1136/ard.56.8.470 PMC1752430

[8] Fong SY Tan JL Septic arthritis after arthroscopic anterior cruciate ligament reconstruction. Ann Acad Med Singapore 2004;33:228–34 15098639

[9] Kusmanova SI Atanassov AN Andreev SA Solakov PT Minor and major complications of arthroscopic synovectomy of the knee joint performed by rheumatologist. Folia Medica 2003;45:55–9 15366667

[10] Indelli PF Dillingham M Fanton G Schurman DJ Septic arthritis in postoperative anterior cruciate ligament reconstruction. Clin Orthopedics Rel Res 2002;398:182–8 10.1097/00003086-200205000-00026 11964649

[11] McAllister DR Parker RD Cooper AE Recht MP Abate J Outcome of postoperative septic arthritis after anterior cruciate ligament reconstruction. Am J Sport Med 1999;27:562–70 10.1177/03635465990270050301 10496570

[12] Kane D Veale DJ FitzGerald O Reece R Survey of arthroscopy performed by rheumatologists. Rheumatology 2002;41:210–15 11886972 10.1093/rheumatology/41.2.210

[13] Hollander JL Intrasynovial corticosteroid therapy in arthritis. Md State Med J 1969;19:62–6 5441259

[14] Gray RG Tenenbaum J Gottlieb NL Local corticosteroid injection treatment in rheumatic disorders. Semin Arthritis Rheum 1981;10:231–54 6787706 10.1016/0049-0172(81)90001-9

[15] Esterhai JL Jr Gelb I Adult septic arthritis. Orthopedic Clin NA 1991;22:503–14 1852426

[16] Le Dantec L Maury F Flipo RM Laskri S Cortet B Duquesnoy B Peripheral pyogenic arthritis. A study of one hundred seventy-nine cases. Revue du Rhumatisme 1996;63:103–10 8689280

[17] Östensson A Geborek P Septic arthritis as a non-surgical complication in rheumatoid arthritis: relation to disease severity and therapy. Br J Rheumatol 1991;30:35–8 1991215 10.1093/rheumatology/30.1.35

[18] Albert C Brocq O Gerard D Roux C Euller-Ziegler L Septic knee arthritis after inra-articular hyaluronate injection. Two case reports. J Joint Bone Spine 2006;73:205–7 10.1016/j.jbspin.2005.03.005 16046172

[19] Goldenberg DL Infectious arthritis complicating rheumatoid arthritis and other chronic rheumatic disorders. Arthritis Rheum 1989;32:496–502 2650687 10.1002/anr.1780320422

[20] Cawley PJ Morris IM A study to compare the efficacy of two methods of skin preparation prior to joint injection. Br J Rheumatol 1992;31:847–8 1458292 10.1093/rheumatology/31.12.847

[21] Charalamobous CP Rryfonidis M Sadiq S Hirst P Paul A Septic arthritis following intra-articular steroid injection of the knee: a survey of current practice regarding antiseptic technique used during intra-articular steroid injection of the knee. Clin Rheumatol 2003;22:386–90 14677011 10.1007/s10067-003-0757-7

[22] Mimoz O Karim A Mercat A Cosseron M Falissard B Parker F Chlorhexidine compared with povidone-iodine as skin preparation before blood culture: a randomized, controlled trial. Ann Int Med 1999;131:834–7 10610628 10.7326/0003-4819-131-11-199912070-00006

[23] American College of Rheumatology Subcommittee on Rheumatoid Arthritis Guidelines Guidelines for the management of rheumatoid arthritis: 2002 update. Arthritis Rheum 2002;46:328–46 11840435 10.1002/art.10148

[24] Yagupsky P Bar-Ziv Y Howard CB Dagan R Epidemiology, etiology and clinical features of septic arthritis in children younger that 24 months. Arch Pediatr Adolesc Med 1995;149:537–40 7735407 10.1001/archpedi.1995.02170180067010

[25] Wang CL Wang SM Yang YJ Tsai CH Liu CC Septic arthritis in children: relationship of causative pathogens, complications and outcome. J Microbiol Immunol Infect 2003;36:41–6 12741732

[26] Goergens ED McEvoy A Watson M Barrett IR Acute osteomyelitis and septic arthritis in children. J Pediatr Child Health 2005;41:59–62 10.1111/j.1440-1754.2005.00538.x 15670227

[27] Peltola H Vahavanen V Acute purulent arthritis in children. Scand J Infect Dis 1983;15:75–80 6844880 10.3109/inf.1983.15.issue-1.12

[28] Kunnamo I Kallio P Pelkonen P Incidence of arthritis in urban Finnish children. A prospective study. Arthr Rheum 1986;29:1232–8 3768057 10.1002/art.1780291008

[29] Gavet F Tournadre A Soubrier M Ristori JM Dubost JJ Septic arthritis in patients aged 80 and older: a comparison with younger adults. J Am Geriatr Soc 2005;53:1210–13 16108940 10.1111/j.1532-5415.2005.53373.x

[30] Joseph ME Sublett KL Katz AL Septic arthritis in the geriatric population. J Oklahoma Med Assoc 1989;82:622–5 2621497

[31] Vincent GM Amirault JD Septic arthritis in the elderly. Clin Orthop Relat Res 1990;251:241–5 2295180

[32] McGuire NM Kauffman CA Septic arthritis in the elderly. J Am Geriatr Soc 1985;33:170–4 3973335 10.1111/j.1532-5415.1985.tb04887.x

[33] Goldenberg DL Septic arthritis. Lancet 1998;351:197–202 9449882 10.1016/S0140-6736(97)09522-6

[34] Torre IG Advances in the management of septic arthritis. Rheum Dis Clin NA 2003;29:61–73 10.1016/s0889-857x(02)00080-7 12635500

[35] Tarkowski A Infectious arthritis. Best Pract Res Clin Rheum 2006;20:1029–44 10.1016/j.berh.2006.08.001 17127195

[36] Mathews CJ Kingsley G Field M Jones A Weston VC Phillips M Management of septic arthritis: a systematic review. Ann Rheum Dis 2007;66:440–5 17223664 10.1136/ard.2006.058909 PMC1856038

